# Rare metastasis to paranasal sinuses from triple-negative breast cancer: A case report and literature review

**DOI:** 10.1097/MD.0000000000008718

**Published:** 2017-11-27

**Authors:** Jie Xiong, Jing Chen, Liduan Zheng, Shengli Yang, Guifang Zhao, Jing Cheng

**Affiliations:** a Cancer Center; b Department of Pathology, Union Hospital, Tongji Medical College, Huazhong University of Science and Technology, Wuhan, Hubei province, China.

**Keywords:** metastasis, paranasal sinuses, triple-negative breast cancer

## Abstract

**Rationale:**

Breast cancer, the most common form of cancer among women, rarely metastases to the head and neck region. To date, there have been only 6 similar cases in the literature, and most patients in these reports had very poor prognosis.

**Patients concerns:**

We report a 61-year-old female presented pain and numbness on the right side of the face 5 years after being diagnosed with triple-negative breast cancer.

**Diagnosis:**

Magnetic resonance imaging (MRI) revealed a tissue mass in the sphenoid sinus. The tissue biopsy confirmed metastasis of breast cancer.

**Intervention:**

The patient received initial chemotherapy and radiotherapy plus 10 cycles of maintenance chemotherapy

**Outcomes:**

The patient got long-term progression-free survival time. The total time to progression was 32 months.

**Lessons:**

Although breast cancer rarely metastasizes to the head and neck region, awareness should be raised when breast cancer patients experience headache or have sinus-related symptoms. Chemotherapy and radiotherapy may be effective to treat paranasal sinus metastasis of triple-negative breast cancer, and patients may achieve long-term survival.

## 1 Introduction

Breast cancer is the most common malignant tumor among Chinese women.^[1]^ The typical breast cancer metastatic sites are regional lymph nodes, bones, lung, liver, and brain. Metastasis to the head and neck region is extremely rare, with only 6 reported cases in the literature.^[2–7]^ In these cases, most patients did not receive any systemic treatment and had a very poor prognosis. Here, we present a recent case of triple-negative breast cancer, the most malignant type of breast cancer, metastasis to the sphenoid sinuses. This patient received initial chemotherapy and radiotherapy plus 10 cycles of maintenance chemotherapy and achieved long-term and progression-free survival. Additionally, we reviewed the current literature regarding the presentation, investigation, diagnosis, management, and prognosis of this rare condition.

## 2 Case report

**Figure 1:**
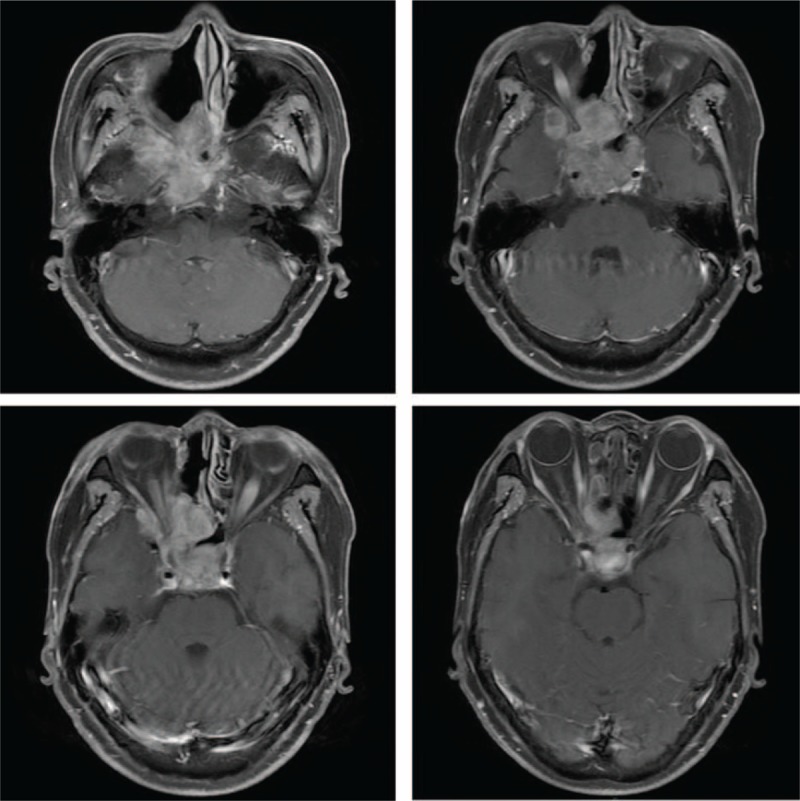
T1-weighted image with contrast enhancement showing a soft tissue mass measuring 5.0 × 4.3 × 6.1 cm in the saddle area and 2 sided sphenoid sinus.

**Figure 2:**
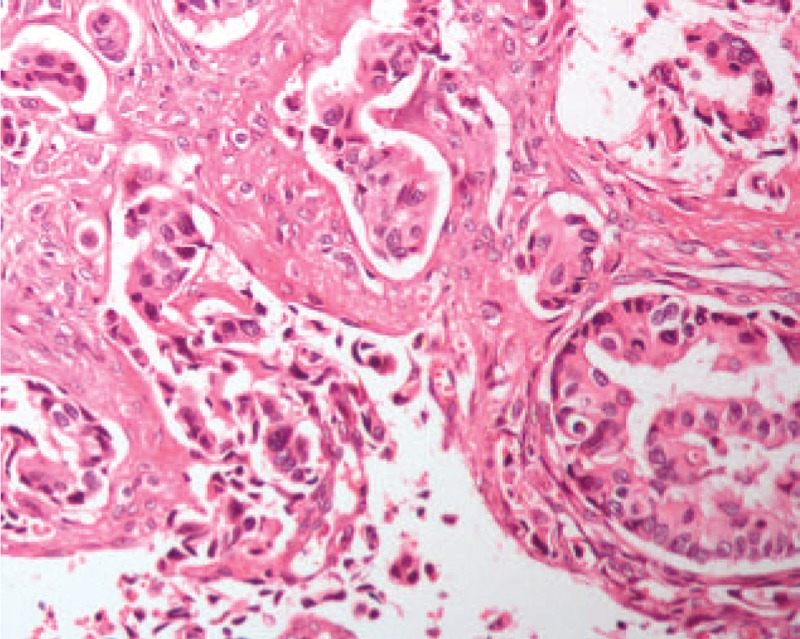
Microscopic appearance of the neoplasm (hematoxylin-eosin, original magnification ×200).

In early 2014, a 61-year-old woman experienced numbness and pain on the right side of her face, accompanied with impaired vision in the right eye. Magnetic resonance imaging (MRI) revealed a soft tissue mass measuring 5.0 × 4.3 × 6.1 cm spreading both sides of the sphenoid sinus, with extension to the ethmoid sinus, cranial base, base of the middle cranial fossa, and the adjacent right temporal lobe parenchyma (Fig. 1). Incisional exploration and biopsy were performed under local anesthesia for an accurate diagnosis. Biopsy results indicated that the mass was a poorly differentiated adenocarcinoma (Fig. 2).

The review of medical history found that the patient was diagnosed with a 35 mm intraductal carcinoma in the left breast in 2009 and treated with radical mastectomy and axillary node clearance. Eleven axillary lymph nodes were removed, and none of them was positive for breast carcinoma. The carcinoma was classified as histological grade 3 and staged as pT2N0M0 according to the Tumor, nodes and metastasis staing system. Further analysis revealed the cancer specimen was negative for estrogen receptor (ER), progesterone receptor (PR), and human epidermal growth factor receptor 2 (HER-2), indicating that the malignancy was triple-negative breast cancer. Following primary mastectomy, the patient underwent chemotherapy with the FAC regimen (fluorouracil, doxorubicin, and cyclophosphamide) for 4 cycles. The patient remained asymptomatic and was free of recurrence during routine follow-up care for 4 years.

The sphenoid sinus specimen was processed for immunohistochemical staining. The results showed the lesion was positive for CK7 and negative for CDX2, CK20, TTF–1, villin, ER, PR, and HER-2. The histology sections of the primary breast lesion of this patient were obtained from the hospital where the patient underwent mastectomy and compared with the sphenoid sinus sections. The specimen from the sphenoid sinus demonstrated similar morphological and immunophenotypic features to the primary breast cancer tissue. This evidence proved that the mass in the sphenoid sinus was metastasized from the primary breast carcinoma. Further examinations, including bone scan and computed tomography (CT) of the chest, abdomen, and pelvic confirmed the absence of other distant metastases.

The patient was treated with the docetaxel and cisplatin (DP) regimen (docetaxel 75 mg/m^2^ and cisplatin 100 mg/m^2^) for 4 cycles, followed by radiotherapy with concurrent weekly 40 mg/m^2^ cisplatin treatment. A total of 54 Gy intensity-modulated radiotherapy (IMRT) was administered to the metastatic sites. After the chemotherapy and radiotherapy, the pain was reduced, and MRI evaluation of the maxillofacial region indicated that the tumor size was stabilized. The patient subsequently received maintenance chemotherapy of a single drug docetaxel (75 mg/m^2^) for 10 cycles. The follow-up MRI examination did not find any radiological progression until October 2016, when the patient was diagnosed with vertebral and liver metastases. The total time to progression was 32 months.

All the procedures were performed after obtaining informed consent, and the patient provided a written informed consent for this case report. A retrospective patient case report which is not conducted with patients to evaluate new medical treatment does not require ethics committee or institutional review board approval according to our guideline.

## 3 Discussion

In this report, we present a rare case of triple-negative breast cancer metastasis to the sphenoid sinuses. Despite being the most prevalent neoplasm in women, metastasis of breast cancer to the head and neck region is rare. Our literature search of similar cases only identified 6 reported cases in the English literature (Table 1). Of these 6 cases, 3 patients received palliative radiotherapy, 1 patient received radiotherapy and oral tamoxifen treatment, and 1 patient received capecitabine therapy. Two patients died in 5 months after diagnosis. The patient received radiotherapy and tamoxifen treatment was diagnosed with luminal A type breast cancer by immunohistochemical staining and survived for 2 years. In general, breast cancer is classified into the 4 molecular subtypes including luminal A, luminal B, triple negative, and HER2 positive. The luminal subtypes typically have good prognosis. All breast cancer patients in the 6 published reports were diagnosed with breast cancer of the luminal subtypes, however, together with metastasis to the head and neck region, most of these patients had poor prognosis. Our case was triple-negative breast cancer, the one associated with the poorest prognosis, but the time to progression was 32 months due to our treatment regimen.

**Table 1:**
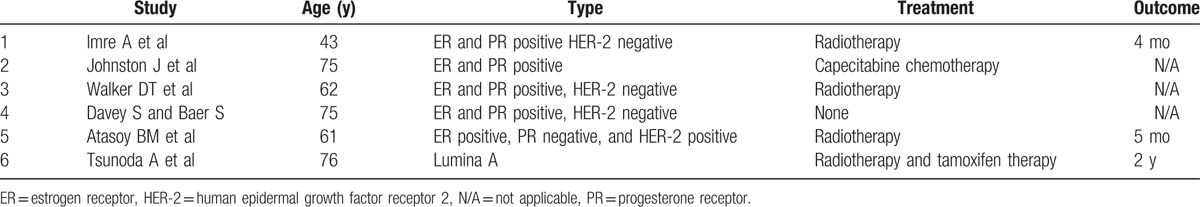
Summary of reported case of breast cancer metastasis to paranasal sinuses.

The etiology of breast cancer metastasis to the head and neck region is largely unknown. Nahum and Bailey^[8]^ proposed that the possible route of hematogenous spread to the sinuses is through the caval venous system, pulmonary circulation, heart, and the arterial vessels of the head and neck. Batson^[9]^ suggested the route is through the vertebral veins and the jugular venous system, which sometimes can reach the skull base. He demonstrated that when the intrathoracic pressure is increased greatly, a retrograde flow from the vertebral venous plexus would form. This retrograde flow may sometimes ascend to as far as the base of the skull. Furthermore, a similar retrograde flow may be observed in the jugular venous system. As there is a rich venous network near the sinuses, these 2 pathways may play a role in the metastasis of cancer cells to these sites.

The clinical symptoms of paranasal sinus metastasis are similar to those seen in rhinosinusitis, which may confuse the diagnosis. A variety of signs and symptoms may be present, including nasal obstruction, epistaxis, and craniofacial pain. Cranial nerve deficits include decreased visual acuity, hearing loss, and eyelid droop. Headache may be the first sign to indicate skull base and meningeal involvement. It may be very difficult to make an accurate diagnosis based on these nonspecific symptoms. Therefore, various diagnostic interventions, such as medical history review, neurological examinations, and advanced imaging techniques, are required for an adequate diagnosis. CT is the initial radiological investigation of choice and should examine the neck, chest, abdomen, and pelvis if a history of breast cancer is noted. MRI is the most powerful tool to examine lymph node involvement and the lesions in the paranasal sinus, skull base, and intracranial regions. Positron emission tomography (PET) scan is potentially useful in detecting earlier lesions, and high SUVmax may correlate with poor prognosis.^[10]^ Tissue biopsy and histopathological analysis are valuable diagnosis if head and neck metastasis is suspected. In the present case, the patient experienced numbness on the right side of the face. MRI revealed a soft tissue mass in the paranasal sinus, and the biopsy of the mass was consistent with metastases from her primary breast carcinoma.

In the published reports of breast carcinoma metastasis to paranasal sinuses, the treatments were generally palliative with the aim of relieving symptoms, and no systemic treatment was adopted in most of these reports.^[2,6]^ Radiotherapy is the standard treatment, and some patients may benefit from chemotherapy or hormone therapy.^[7]^ Surgical resection can be helpful in some patients.^[11]^ In the present case, the patient was unsuitable to receive radical resection because the lesion had extended to the base of the middle cranial fossa and the adjacent right temporal lobe. Because the patient had not previously received Taxol treatment, and platinum agents are usually effective for triple-negative breast cancer, this patient was treated with chemotherapy with the DP regimen for 4 cycles with the aim of shrinking the tumor and of testing its sensitivity to the chemotherapy. The tumor stabilized after chemotherapy, and the patient was treated with further radiotherapy to the metastatic site, with concurrent weekly 40 mg/m^2^ cisplatin to enhance the sensitivity of radiotherapy. The pain was reduced following chemotherapy and radiotherapy, and the patient subsequently received maintenance chemotherapy of a single drug docetaxel for 10 cycles. This treatment did not show any intolerable side effects.

In the 6 reported cases, paranasal sinus and skull-base metastases of breast cancer had very poor prognosis, probably because nasopharynx metastases occurred late in the course of the malignancy.^[12]^ Two patients died in 5 months after diagnosis,^[2,6]^ and the patient with luminal A type breast cancer, as diagnosed with immunohistochemical staining, survived for 2 years,.^[7]^ Our patient had triple-negative breast cancer, which is associated with the poorest prognosis, but no progression over a 32-month period was observed after the diagnosis of metastasis to paranasal sinuses. This suggests that chemotherapy and radiotherapy are effective to treat paranasal sinus metastasis, and docetaxel combined with cisplatin regimen can be the choice of chemotherapy.

## 4 Conclusion

Although breast cancer rarely metastasizes to the head and neck region, awareness should be raised when breast cancer patients present headache or sinus symptoms. Chemotherapy and radiotherapy may be effective to treat paranasal sinus metastasis, and patients can achieve long-term survival. To the best of our knowledge, this is the first report of the metastasis of triple-negative breast cancer to the head and neck region, and the first time that the systemic treatment of this type of cancer is discussed in the literature.
